# Azaacenes Based Electroactive Materials: Preparation, Structure, Electrochemistry, Spectroscopy and Applications—A Critical Review †

**DOI:** 10.3390/ma14185155

**Published:** 2021-09-08

**Authors:** Kamil Kotwica, Ireneusz Wielgus, Adam Proń

**Affiliations:** 1 Institute of Physical Chemistry, Polish Academy of Sciences, Kasprzaka 44/52, 01-224 Warszawa, Poland; 2 Faculty of Chemistry, Warsaw University of Technology, Noakowskiego 3, 00-664 Warszawa, Poland; iwielgus@ch.pw.edu.pl (I.W.); apron@ch.pw.edu.pl (A.P.)

**Keywords:** azaacenes, synthesis, functionalization, redox properties, devices

## Abstract

This short critical review is devoted to the synthesis and functionalization of various types of azaacenes, organic semiconducting compounds which can be considered as promising materials for the fabrication of n-channel or ambipolar field effect transistors (FETs), components of active layers in light emitting diodes (LEDs), components of organic memory devices and others. Emphasis is put on the diversity of azaacenes preparation methods and the possibility of tuning their redox and spectroscopic properties by changing the C/N ratio, modifying the nitrogen atoms distribution mode, functionalization with electroaccepting or electrodonating groups and changing their molecular shape. Processability, structural features and degradation pathways of these compounds are also discussed. A unique feature of this review concerns the listed redox potentials of all discussed compounds which were normalized vs. Fc/Fc^+^. This required, in frequent cases, recalculation of the originally reported data in which these potentials were determined against different types of reference electrodes. The same applied to all reported electron affinities (EAs). EA values calculated using different methods were recalculated by applying the method of Sworakowski and co-workers (Org. Electron. 2016, 33, 300–310) to yield, for the first time, a set of normalized data, which could be directly compared.

## 1. Acenes vs. Azaacenes—Generalities

In the past two decades significant research effort in the domain of organic semiconductors was directed towards acenes and azaacenes [1,2,3,4]. Several types of acenes were tested as semiconducting active layers in p-channel organic field effect transistors (OFETs). Interesting properties of these compounds resulted from a relatively low value of their ionization potential which facilitated the injection of holes to the active layer in the transistor operating conditions. Investigated acenes readily crystallized due to planarity and frequent high symmetry of their molecules, yielding good quality single crystals or highly crystalline thin layers when deposited on an appropriate substrate. Some of them exhibited extremely high value of the hole mobility. For example, hole mobility of 40 cm^2^/Vs was reported for single crystals of rubrene (5,6,11,12-tetraphenyltetracene) [5].

From the point of view of technological applications, limited stability of acenes towards oxidative degradation constitutes the main drawback of these semiconductors. This especially applies to higher acenes consisting of several aromatic rings. Formation of carbonyl groups as a result of oxidative degradation perturbs the conjugation in individual molecules and induces disorder in their supramolecular organization, resulting in a drastic decrease of the charge carriers mobility [6].

Acenes cannot be used as semiconductors transporting electrons in any organic electronic devices, including n-channel OFETs, because their LUMO level is too high, implying low electron affinity (EA) values and by consequence very difficult electron injection [7,8].

Azaacenes (*N*-heteroacenes) are derivatives of acenes in which a fraction of carbon atoms are substituted by more electronegative nitrogen atoms. This substitution induces significant changes in their electronic, redox and spectroscopic properties. Quantum-chemical calculations, and especially those based on Density Functional Theory (DFT), constitute a powerful tool for the elucidation of the effect of N/C ratio and the N atoms distribution mode on the electronic and redox properties of azaacenes. This approach allows for the determination of their HOMO and LUMO levels as well as their ionization potentials (IPs) and electron affinities (EAs) in vacuum or in a given solvent. The latter can be confronted with experiments consisting of chronovoltamperometric determination of IP and EA. Even though the experimental values may sometimes differ from the calculated ones, especially in the case of EA, the observed trends usually remain the same.

As demonstrated by DFT calculations, for linear acenes and azaacenes the positions of HOMO and LUMO levels are strongly dependent on the number of aromatic rings in the molecule. In particular, LUMO level decreases and HOMO level increases with increasing length of the molecule [9]. If acene and azaacene of the same number of rings are compared, both HOMO and LUMO levels are lowered with increasing N/C ratio. For example, LUMO and HOMO levels of pentacene are located at −2.7 eV and at −4.6 eV while in the corresponding diazapentacene (naphtho[2,3-*b*]phenazine) these levels are lowered to −3.3 eV and −5.1 eV, respectively. In tetraazapentacene (quinoxalino[2,3-*b*]phenazine) they are further lowered to −3.6 eV and −5.3 eV [10]. Moreover, in the case of azaacenes of the same number of rings and the same number of nitrogen atoms, the HOMO and LUMO energies are sensitive to the nitrogen atoms distribution. Nitrogens located in terminal rings have less pronounced effect on the energies of LUMO and HOMO than those from inner rings. Similar trends are observed in the case of azahexacenes [11,12]. HOMO and LUMO energies as well as closely related to them through the Koopmans theorem IP and EA values determine electronic, spectroscopic and redox (electrochemical) properties of azaacenes.

Trends evidenced by DFT calculations are corroborated by experiment. In Table 1 experimentally determined redox potentials, electron affinities and optical band gaps are compared for the shortest acenes (naphthalene and anthracene) and their azaacene analogues containing increasing number of nitrogen atoms.

As evidenced from these data, the band gap (E_g opt_) decreases with increasing N/C ratio whereas the electron affinity (EA) increases in full accordance with DFT data. In particular, the reduction potential of naphthalene is significantly lower than the corresponding potentials of its aza analogues, i.e., quinoline and quinoxaline. The same applies to anthraquinone and its aza-derivatives. Note that for the same number of aromatic rings (three) EA increases in a series: acridine (1N), phenazine (2N), pyrazinoquinozaline (4N). This trend is maintained in azaacenes of higher molecular mass and different molecular shape.

Further tuning of electronic, optical and redox properties of azaacenes is possible by introduction of substituents of electrodonating or electroaccepting properties. Solution processability of azaacenes, frequently very difficult or even nonexistent, can also be improved by attaching appropriate solubilizing groups to the conjugated core.

Azaacenes are aromatic compounds, however their expected aromaticity should be lower than that of the corresponding acenes. It is instructive to compare aromaticities of the simplest aromatic and aza-aromatic compounds such as benzene, pyridine and three diazines, i.e., 1,2-diazine (pyridazine), 1,3-diazine(pyrimidine) and 1,4-diazine (pyrazine). Pyridine, pyrazine and pyrimidine are highly aromatic. Their HOMA (Harmonic Oscillator Model of Aromaticity) indices exceed 0.99. The only exception is pyridazine (1,2-diazine) whose HOMA index is significantly lower and equals to 0.911 [23]. This pronounced decrease of aromaticity is caused by the presence of two nitrogen atoms at adjacent positions in the ring, forming an N-N bond. Acenes and azaacenes reflect this trend indicating higher aromaticity of the former. It should also be noted that their HOMA indices are in line with those derived from NICS (Nucleus-Independent Chemical Shifts) [24].

Redox properties of azaacenes are inherently associated with their chemical constitution involving aromatic core and eventually the presence of electron donating (electron accepting) substituents. Redox properties of acenes and azaacenes are most frequently experimentally determined by cyclic voltammetry (cv) or differential pulse voltammetry (dpv). These data are usually available in literature; however, the determined redox potentials are expressed versus different reference electrodes which makes their comparison difficult. For comparative reasons all redox potentials presented in this review are expressed vs. ferrocene/ferrocenium couple (Fc/Fc^+^). This means that all redox potentials reported versus different than Fc/Fc^+^ references were recalculated by the authors of this review. This approach allows for direct comparison of the redox properties of all azaacenes discussed here.

The electrochemically measured redox potentials can in principle be used for the determination of the solid state ionization energies such as ionization potential (IP) or electron affinity (EA) of electroactive molecules, i.e., parameters which are crucial for the majority of organic semiconductors applications. This approach requires, however, some comments. IP and EA can be directly measured on thin organic semiconductor films using ultraviolet photoelectron spectroscopy (UPS) and inverse photoelectron spectroscopy (IPES), respectively. Electrochemical experiments are carried out in solution, thus in this respect they do not provide direct access to IP and EA. In the majority of papers IP and EA are calculated from the electrochemical data using the following equations:

EA = eE_red_ + C (1)

In addition:

IP = eE_ox_ + C (2)

where E_red_ and E_ox_ stand for the first reduction potential and the first oxidation potential of the studied molecule, respectively; C is the absolute energy of the Fc/Fc^+^ redox couple in the electrolyte solution.

The value of C is still the subject of debate and its value can range from 4.8 eV to 5.1 eV in various reports (see for example discussion in [25]). Applications of different values of C makes the EA and IP values, reported in various papers, difficult to compare. Moreover, as pointed out by Sworakowski et al. in a series of papers [22,26,27], the EA and IP values derived from electrochemical experiments carried out in solutions and calculated using Equations (1) and (2) may significantly differ from those determined for thin films by UPS and IPES, thus not giving direct access to solid state ionization energies. These authors, analyzing all available electrochemical and photoelectron spectroscopy data, pointed out that a clear correlation exist between the electrochemically determined redox potentials and experimentally determined IP and EA values which can be expressed by the following equations:

EA = 1.18 × eE_red_ + 4.79 [eV] (3)

In addition:

IP = 1.15 × eE_ox_ + 4.83 [eV] (4)

Again, for comparative reasons we used Equations (3) and (4) for the calculations of EA and IP values for all azaacenes reported in this review.

Depending on N/C ratio azaacenes can show either n-type, p-type or ambipolar character in the field effect transistor configuration. The latter means that they can transport either holes or electrons, depending on the electrodes’ polarity. For high N/C ratio they lose their ambipolar character and are used as electrons transporting materials in n-channel field effect transistors. This is expected, since a significant increase of EA of these compounds facilitates the stabilization of radical anions formed temporarily upon the injection of electrons from the source electrode [28,29]. An increase of IP of azaacenes as compared to acenes leads to a significant improvement in their resistance against oxidative degradation, making the formation of carbonyl groups and/or degradative dimerization more difficult [30]. Azaacenes find their application as organic semiconductors not only in electronics but also in optoelectronics as electroluminophores in organic light emitting diodes (OLEDs) [31,32,33,34] and in photovoltaics as acceptors in non-fullerene organic photovoltaic cells [35,36,37].

Research on electroactive azaacenes is principally focused on three types of compounds: (i) linear which can be considered as analogues of the corresponding linear acenes; (ii) more condensed azaacenes containing pyrene-type segments; (iii) star-shaped azaacenes. This classification should be considered as somehow fuzzy since large number of azaacenes exist which cannot be attributed to any of these groups or they are on the borderline of two groups.

## 2. Synthesis of Azaacenes

First syntheses of azaacenes were reported at the end of 19th century by Fischer and Hepp [38,39]. These research efforts were then continued through the 20th century and significantly intensified in recent years [40,41].

Initially elaborated methods of azaacenes synthesis were based on reactions of o-dihalogeno (or *o*-dihydroxyaryl) aromatic compounds with aromatic o-diamines. The condensation reactions were usually carried out at temperatures exceeding melting points of the substrates, typically in the range of 130–160 °C, but sometimes at temperatures exceeding 200 °C. In these reaction conditions the target azaacenes (or dihydroazaacenes) could be obtained in reasonable yields. The above outlined simple preparation methods are exploited even nowadays, especially in cases where the substrates and the product are insufficiently soluble in conventional organic solvents [42]. Low reactivity of aryl o-diamines can be considered as a somehow weaker point of this synthetic pathway [43].

Over the years, the condensation methods of azaacenes preparation have been constantly modified and improved. In particular, application of protic compounds as solvents or catalytic admixtures led to a significant lowering of the reaction temperature to values below 100 °C (in selected cases even below 50 °C), while retaining high reaction yields [44,45,46].

Condensation of aromatic *o*-diamines with *o*-dihydroxy derivatives of aryl compounds carried out in mild conditions yields dihydroazaacenes whereas the condensation with o-diketones results in azaacenes. In the case of substrates containing hydroxyl groups in addition to keto ones, as in the synthesis with the use of dihydroxybenzoquinone or oxalic acid, dihydroxy derivatives of azaacenes are obtained in the first step, then dihydroazaacenes in the second one, which finally have to be oxidized to tetraazaacenes [45,46].

Optimization of *o*-diketone condensation with *o*-diamines (or their salts) leads to various pyrazine derivatives which can be obtained in good yields. Various azaacenes of extended structures were obtained at room temperatures using acetic acid in chloroform [47]. It should be noted that azaacenes of very diversified nitrogen atoms distribution can be obtained via condensation of amines with ketones. For example, Zhu et al. [48] prepared phthalazine derivatives in which nitrogen atoms occupied adjacent positions.

**Figure 1:**
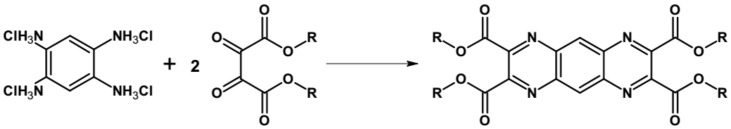
Preparation of 1,4,5,8-tetraazaanthracene containing four solubilizing ester groups, R = Me, Et or Bu.

An interesting procedure of the synthesis of 1,4,5,8-tetraazaanthracene functionalized with four solubility-inducing ester groups was reported [49]. In this method, protected substrate, namely 1,2,4,5–benzene tetraamine tetrahydrochloride, upon deprotection in the reaction medium with a weak base (sodium acetate), reacted with dioxosuccinic acid ester to yield the desired product (see Figure 1). The reaction yield was 50%. Further modification through trans-esterification allowed for introduction of R groups different from the initial ones.

**Figure 2:**
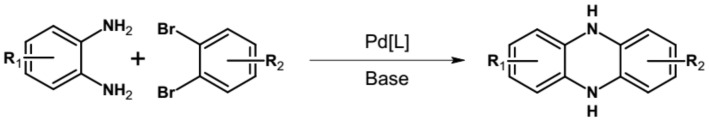
General scheme of Buchwald-Hartwig coupling, Br or Cl.

The most general approach to the synthesis of hydroazaacenes, which upon oxidation can be transformed into azaacenes, is based on the Buchwald-Hartwig coupling reaction. This especially applies to dihydrophenazines but also to longer hydroazaacenes in a form of tetrahydroazaacenes or hexahydroazaacenes [50,51,52]. Buchwald-Hartwig coupling allows for the formation of C-N bonds in significantly milder conditions as compared to other methods, facilitating in this manner the introduction to the conjugated core substituents of different type. In the simplest version of the discussed reaction aryl *o*-diamines are reacted with aryl *o*-dihalides in the presence of a palladium complex catalyst and a base. Ligands in the palladium complex are appropriately selected, depending on the solvent used (see Figure 2).

An instructive approach to the preparation of azaacenes via Buchwald-Hartwig coupling can be found in reference [43].

Reaction yields in Buchwald-Hartwig coupling depend on the nature of substituents in the substrates [50,51]. In particular, in the synthesis of dihydrophenazine derivatives, a decrease of the reaction yield was observed if substituents showing electron accepting properties were introduced to 2,3-dichloroquinoxaline. Functionalization of diamine substrates had less pronounced effect on the obtained reaction yields. Similar tendency was also observed in the synthesis of fluoro derivatives of azapentacenes which were obtained with a significantly lower yields as compared to their non-fluorinated counterparts [53].

One has to be aware of the fact, that in this reaction two C-N bonds have to be formed in the ortho positions with respect to each other. This may induce steric problems which then, together with lower reactivity of *o*-dihalides containing electron accepting groups, may in some cases result in lowering the reaction yield [54].

In view of these briefly discussed shortcomings of Buchwald-Hartwig coupling in the synthesis of azaacenes containing strongly electron accepting substituents, alternative approaches were proposed. Schwaben et al. [53] obtained fluoro-substituted diazapentacenes from aromatic *o*-diamines and perfluorinated bezene or naphthalene using nucleophilic substitution (S_N_Ar). In the first step the treatment with NaH or with sodium bis(trimethylsilyl) amide at RT resulted in the formation of a C-N bond. In the second step, carried out at elevated temperature for extended time, dihydropyrazine ring closure occurred in the presence of a weak base (i-Pr_2_EtN) to yield the target tetra-fluorodiazatetracene. Comparative studies of Buchwald-Hartwig coupling and nucleophilic substitution showed that in some cases the latter resulted in better yields and enabled syntheses of azaacene derivatives unachievable by the former.

In the preparation using Buchwald-Hartwig coupling or related processes, hydroazaacenes are frequently obtained as intermediate products, which have to be then oxidized to yield the target azaacenes. Selection of an appropriate oxidant is of crucial importance in this case. Manganese dioxide (MnO_2_) in chloroform or dichloromethane is one of the most popular oxidizing agents used in the preparation of various types of diazaacenes or tetraazaacenes containing deactivating halo-, nitro- and other substituents [50,52,55]. It was also used in the synthesis of hexaazaacenes [51]. Other oxidants were also applied with success such as *N*-bromoimide of succinic acid, 2-iodobenzoic acid, copper (II) acetate, potassium dichromate (VI), pyridine dichromate (VI), pyridine chlorochromate (VI), 2,3-dichloro-5,6-dicyano-*p*-benzoquinone, chloranil and lead (IV) dioxide. PbO_2_ readily converts hydroazaacenes into azaacenes but in the same time it does not oxidize alkylsillyl groups which are frequently attached to the azaacene core with the goal to improve their solution processability and making them more compatible upon deposition on silicium substrates in hybrid organic/inorganic electronic devices [56]. Oxidation of hydroazaacenes to azaacenes has to be performed with caution because in the case of azaacenes of low N/C ratios, too strong oxidants may result in the formation of benzoquinone-type units, significantly worsening semiconducting properties of these compounds.

An interesting procedure of the preparation of azaacenes was proposed by Raju et al. [57]. These authors developed one-pot synthesis of benzophenazine and its derivatives by reacting benzoxepine-4-carboxylates with benzene-1,2-diamines, in the presence of Bi(OTf)_3_ catalyst. The reaction, carried out under relatively mild conditions resulted in very good yields.

Exploiting appropriate catalytic systems, it is possible to prepare azaacene derivatives in which the distribution of nitrogen atoms differs from that characteristic of the 1,4-pyrazine unit. Reference [58] can be considered here as an instructive example. These authors described the application of nickel-based catalysts in the synthesis of quinazoline derivatives containing either aryl or alkyl substituents in position 2. In this procedure benzylamine with unoccupied *ortho* position was reacted with nitryl group containing benzene, pyridine or another appropriate aromatic reagent. The resulting azaacene was substituted in position 2 with either aliphatic or aromatic substituent originating from the nitryl derivative. This promising method should lead to quinazoline derivatives of more extended aromatic structure.

**Figure 3:**
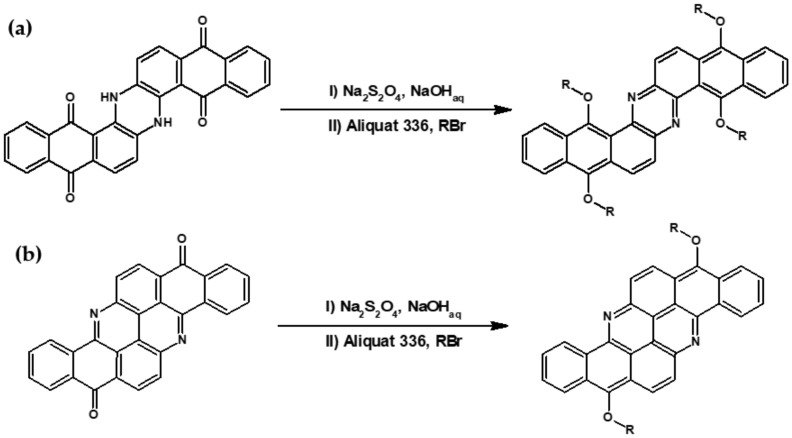
Preparation of: (**a**) tetraalkoxydinaphtho[2,3-*a*: 2′,3′-*h*]phenazine from 6,15-dihydrodinaphtho-[2,3-*a*:2′,3′-*h*]phenazine-5,9,14,18-tetraone (flavanthrone) R = *n*-alkyl; (**b**) 8,16-dialkoxybenzo[*h*]benz[5,6]acridino[2,1,9,8-*klmna*]acridines from benzo[*h*]benz[5,6]acridino[2,1,9,8-*klmna*]acridine-8,16-dione. R = *n*-alkyl group.

Kotwica et al. [59,60] developed a very simple method of the preparation of phenazine derivatives. These authors demonstrated that indanthrone (6,15-dihydrodinaphtho-[2,3-*a*:2′,3′-*h*]phenazine-5,9,14,18-tetraone) an old, nitrogen containing insoluble dye can be converted into a solution processable azaacene, namely tetraoctyloxydinaphtho[2,3-*a*: 2′,3′-*h*]phenazine, in a simple one-pot process consisting of carbonyl groups reduction with sodium dithionite, followed by *o*-alkylation under phase transfer catalysis conditions (see Figure 3a). Continuing their research on nitrogen-containing vat dyes as substrates for the preparation of azaacenes Kotwica et al. [61,62] prepared a series of solution processable fused azaacenes, namely 8,16-dialkoxybenzo[*h*]benz[5,6]acridino[2,1,9,8-*klmna*]acridines from flavanthrone (benzo[*h*]benz[5,6]acridino[2,1,9,8-*klmna*]acridine-8,16-dione)—an almost forgotten dye (see Figure 3b).

**Figure 4:**
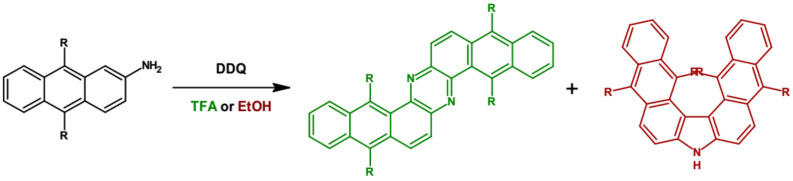
Preparation of tetraalkyldinaphtho[2,3-*a*: 2′,3′-*h*]phenazine from dialkyl-substituted 2-aminoanthraquinone via oxidation with 2,3-dichloro-5,6-dicyano-1,4-benzoquinone (DDQ). R = (triisopropylsilyl)ethynyl [63].

Very similar phenazine derivatives were obtained by oxidation of dialkyl-substituted 2-aminoanthraquinone with 2,3-dichloro-5,6-dicyanobenzenequinone [63]. Depending on the solvent used, two products in varying proportions were obtained: Z-shaped phenazine derivative and V-shaped carbazole one (Figure 4). Admixture of small quantities of trifluoroacetic acid (TFA) to the reaction mixture resulted in a significant increase of the reaction yield with almost 100% selectivity towards phenazine derivatives.

**Figure 5:**
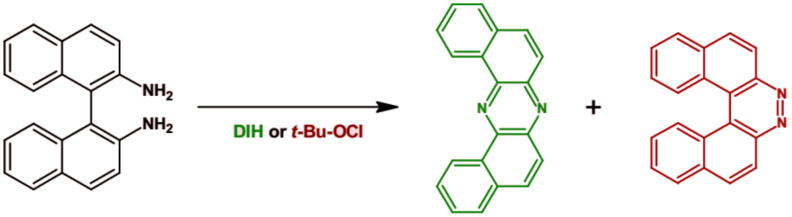
Synthesis of dibenzo[*a*,*j*]phenazine and diaza[5]helicene by oxidation of 1′1-binaphthalene-2,2′-diamine [64].

An interesting procedure of the preparation of phenazine derivatives was proposed by Takeda et al. [64]. Using different oxidizing agents 1,1′-binaphthalene-2,2′-diamine was oxidized to two products: U-shaped phenazine derivative and diaza[5]helicene (see Figure 5). The highest selectivity towards dibenzo[*a*,*j*]phenazine (77%) was obtained when 1,3-diiodo-5,5-dimethyl-2,4-imidazolidinedione (DIH) was used as an oxidant.

**Figure 6:**
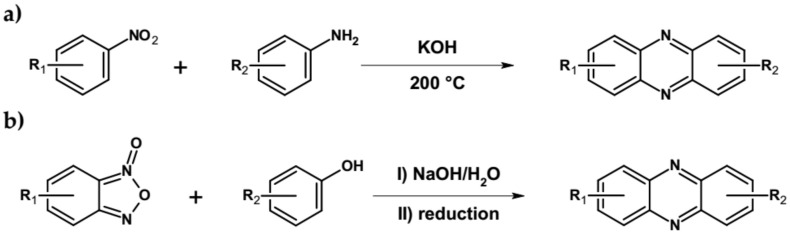
Synthesis of azaacenes via (**a**) Wohl-Aue and (**b**) Beirut reactions.

Other, possible methods of azaacenes preparation should also be mentioned. This involves, among others, base-catalyzed condensation of anilines with nitrobenzenes (Wohl-Aue reaction, see Figure 6a) [65] and dehydrative condensation of benzo[1,2,5]oxadiazole-1-oxide with various nucleophiles (Beirut reaction, Figure 6b) [66].

The above discussed reactions have to be carried out at elevated temperatures, which severely limits their use in the syntheses of less thermally stable azaacenes derivatives.

## 3. Spectroscopic and Electrochemical Properties of Linear Azaacenes

**Figure 7:**
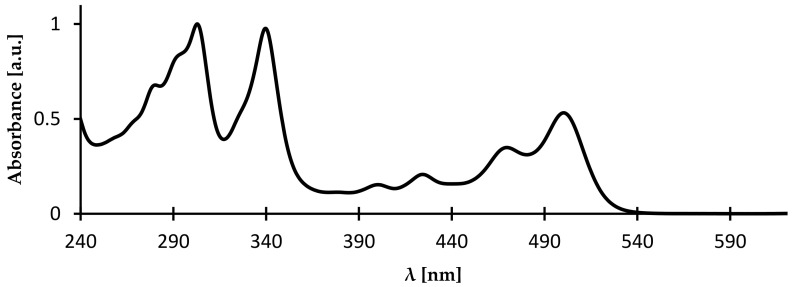
UV-vis spectrum of 5,10,15,18-tetraoctyloxy-8,17-dihydronaphtho[2,3-*a*:2′,3′-*i*]phenazine [67].

UV-vis-NIR spectra of azaacenes are rich. Strong bands in the UV or visible part of the spectrum are accompanied by a less intensive band in the visible or near infrared part of the spectrum. The latter has a strongly vibronic character with the 0–0 transition being the most intensive (see Figure 7). This least energetic band undergoes increasing bathochromic shift with increasing molecular mass of azaacene, i.e., with number of aromatic rings in the molecule.

**Figure 8:**
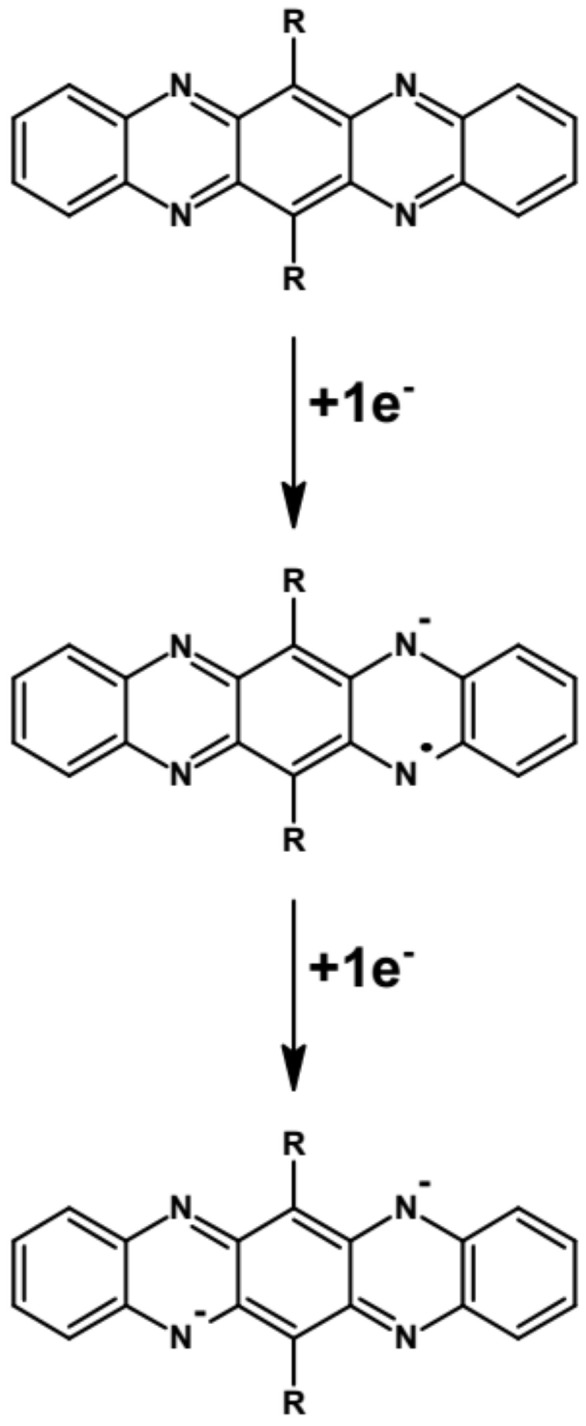
Formation of a radical anion and a spinless dianion in a two-step reduction of disubstituted tetraazapentacene—quinoxalino[2,3-*b*]phenazine. R = (triisopropylsilyl)ethynyl group.

As evidenced by cyclic voltammetry, azaacenes undergo either one-step or two-step reversible or quasi-reversible reduction at relatively high potentials (vs. Fc/Fc^+^). This is associated with their high EA value. In the two-step reduction process radical anions are formed in the first step which are then transformed to spinless dianion in the second one. In Figure 8 these processes are depicted using disubstituted tetraazapentacene (quinoxalino[2,3-*b*]phenazine) as an example.

Radical anions generation can be evidenced in an EPR spectroelectrochemical experiment or through generation of radical anions directly in the EPR tube, using an appropriate chemical reducing agent [68]. Chemical or electrochemical reduction of azaacenes result in the formation of new UV-vis-NIR bands which can be considered as diagnostic of either the radical anion or spin-less dianion states. By combining UV-vis-NIR and EPR spectroelectrochemistry with cyclic voltammetry it is therefore possible to unequivocally attribute these bands to a given reduction state. To the contrary, oxidation of azaacenes is irreversible and starts at rather high potentials, reflecting their higher IP as compared to acenes.

**Figure 9:**
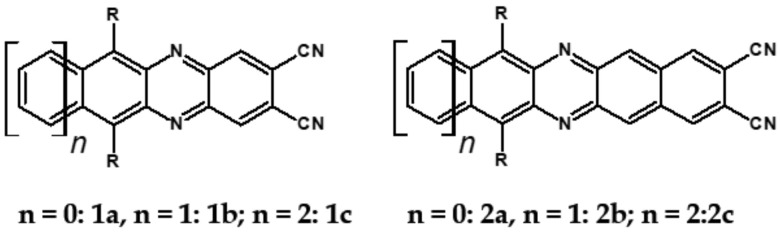
Two series of linear azaacenes synthesized with the goal to investigate the effect of number of rings and electron accepting substituents on their optical and electrochemical properties [69]. R = (triisopropylsilyl)ethynyl group.

As already stated, the first reduction potential of azaacenes can be significantly increased and the band gap narrowed, as a result of two main factors or a combination of them: (i) increased number of aromatic rings and (ii) functionalization with electron withdrawing substituents. These effects were discussed in detail in reference [69]. It is instructive to follow the changes of the optical and redox properties in two series of azaacenes depicted in Figure 9. These diazaacenes in series 1 and 2 differed in the number of aromatic rings. Their terminal rings were substituted with two cyano groups. Compounds of series 2 were systematically longer by one ring. The effect of the cyano groups on the LUMO level lowering was evidenced by a significant shift of their first reduction formal potential (E_1/2_) as compared to their non-cyanated analogues [70]. In particular E_1/2_ of **1a** was shifted by 600 mV to −1.1 V with respect to the same potential of its non-cyanated analogue (−1.7 V). Very similar effects were observed for **1b**, **1c**, **2a** and **2b**. This implied significantly increased EA values. They are listed in Table 2 together with E_1/2_ and optical band gaps determined from the positions of the 0–0 transitions.

The effect of the increase in the number of rings is also evident by comparing E_1/2_, EA and E_opt_ values within one series. For example, EA of **1c** increased by 0.47 eV with respect to that measured for **1a** whereas the band gap decreased to 1.49 eV, i.e., by 0.83 eV. **1c**, **2b** and **2c** can be considered as low band gap, easily reducible semiconductors. Such materials are highly desirable in organic electronics and photovoltaics.

**Figure 10:**
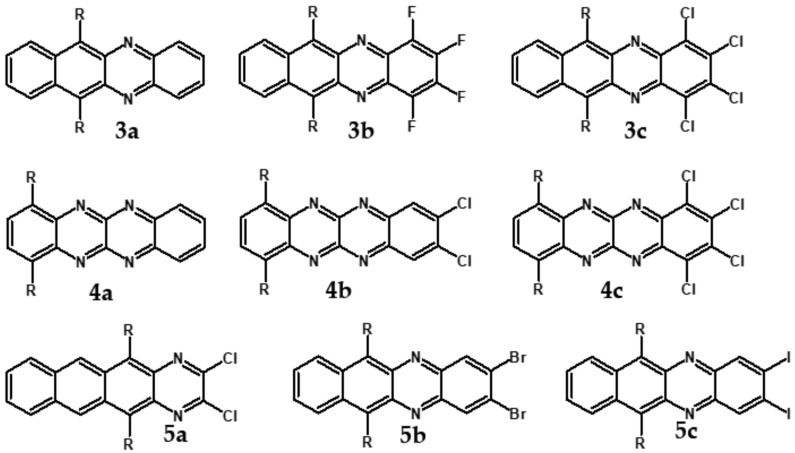
Selected halogen derivatives of di- and tetraazaacenes [50,71]. R = (triisopropylsilyl)ethynyl group.

Halogens are also popular terminal ring substituents lowering the band gap of azaacenes and facilitating their reduction. In Figure 10 two series of compounds are depicted namely di- and tetraazaacenes differently halogenated in their terminal ring. Their spectroscopic parameters are collected in Table 3.

These data indicate that the introduction of halogen substituents induces a bathochromic shift of the 0–0 transition. This effect is more pronounced in the emission spectra since substitution with halogens leads to increasing Stokes shift, especially in the case of heavier substituents such as Br and I [71].

The reduction potential of halogen-free diazatetracene (**3a**) is −1.2 V vs. Fc/Fc^+^ [50]. Introduction of halogen substituents to its terminal ring results in an increase of this potential in the case of **3b** and **3c**. Tetraazaacenes **4a**, **4b** and **4c** undergo a two-step reduction with a significant shift of the first reduction potential to −0.7 V and −0.6 V for **4a** and **4b**, respectively [50]. Slightly smaller shift is observed in the case of **4c**.

**Figure 11:**
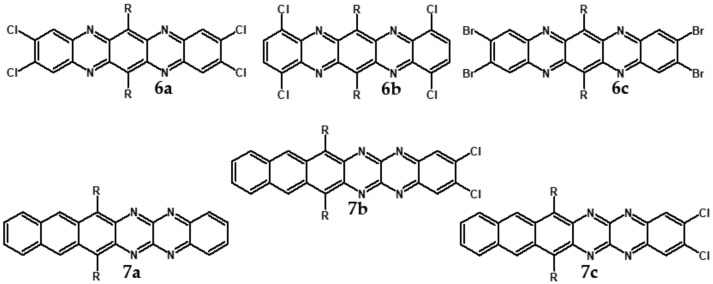
Selected halogen derivatives of tetraazapentacenes [72] and tetraazahexacenes [51]. R = (triisopropylsilyl)ethynyl group.

Electrochemical and spectroscopic properties of longer azaacenes such as tetraazapentacenes [72] or tetraazahexacenes [51] are also very sensitive towards halogen substituents [51,72]. In the case of tetrachloro derivative of bis((tripropan-2-ylsilyl)-ethynyl)tetrachloroquinoxalino[2,3-*b*]phenazine significant differences in the absorption spectra of its two isomers are observed. **6a** (Cl at 2,3,9,10 positions) absorbs at shorter wavelengths as compared to **6b** (Cl at 1,4,8,11 position) (see Table 4). Exchange of chlorine in **6a** for bromine to yield **6c** also induces a bathochromic shift of the absorption band. Halogen derivatives of tetraazahexacenes (**7b** and **7c** see Figure 11) absorb at near infrared part of the spectrum, concomitantly showing high reduction potential and, by consequence, high electron affinity (Table 4).

**Figure 12:**
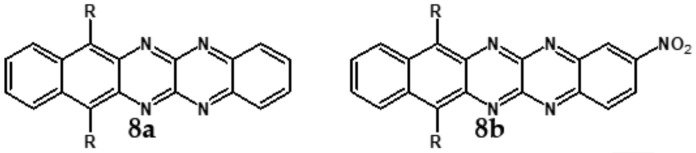
Selected derivatives of tetraazapentecenes [43]. R = (triisopropylsilyl)ethynyl group.

Semiconductors of extremely high EA value can be obtained by introducing nitro groups to the terminal ring of tetraazapentacenes (Figure 12 and Table 4).

**Figure 13:**
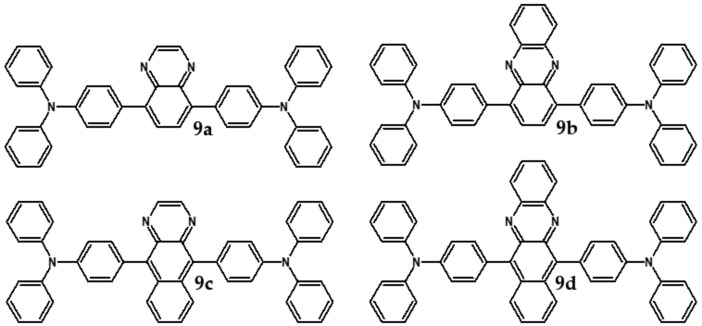
DAD compounds consisting of diazaacene central unit of varying number of aromatic rings and two triphenylamine substituents [73].

Azaacenes with electron donating substituents behave differently. They can be treated as donor-acceptor compounds of DA or DAD type showing in selected cases ambipolarity [73]. Introduction of a donor group results in significant lowering of the ionization potential (IP), thus these derivatives are more readily oxidizable than nonsubstituted azaacenes. A series of DAD compounds consisting of two triphenylamine substituents and diazaacene central group can be considered here as an instructive example [73] (see Figure 13, Table 5).

UV-vis spectra of these compounds are characterized by a distinct band of charge transfer (CT) character which is being bathochromically shifted with increasing number of aromatic rings in the central azaacene unit. The same trend is observed in their emission spectra. These derivatives undergo a two-step oxidation at relatively high potentials. These two oxidations are related to consecutive removal of two electrons from two triphenylamine substituents. The potential of the first oxidation is only weakly dependent on the number of aromatic rings in the central azaacene unit. However, more pronounced changes are observed in the difference between the first and the second oxidation potentials (E_ox2_–E_ox1_) (Table 5). Decreasing (E_ox2_–E_ox1_) value in the case of azaacenes with larger central units can be rationalized by the fact that larger conjugated cores of the central unit better promote delocalization of the positive charge imposed upon the removal of the first electron, facilitating in this manner the removal of an additional electron from the second triphenylamine substituent.

## 4. Supramolecular Organization of Linear Azaacenes

Azaacenes, similarly to acenes, readily crystalize and their supramolecular organization is, to a large extent, determined by π−π stacking of flat molecules. Individual molecules separated by a distance from 0.335 nm to 0.350 nm form slipped studs [69]. Substituents may significantly affect this structural arrangement depending on their size and position in the molecule [69]. Weak noncovalent intermolecular interactions induce sometimes structural dimerization within individual stacking pattern, as in the case of a derivative of phenazine **1a**. In this structural arrangement two intermolecular distances between molecules are observed within the stand. Similar stacking pattern was also observed for diazatetracene containing substituents in its terminal ring (**2a**, [69]). To the contrary, only one intermolecular distance of 0.343 nm within a stack was reported for a different isomer of **1a**, i.e., **1b** [69].

An interesting example of structural engineering of substituted azaacenes can be found in reference [74]. Introduction of long, branched alkoxyl groups to 5,6,13,14-tetraazapentacene resulted in the formation of a smectic-type liquid crystalline phase, stable over a wide temperature range. Liquid crystallinity is advantageous in view of azaacenes application in organic electronics since it facilitates deposition of thin films of desired orientation.

Supramolecular organization of linear azaacenes can be very diversified. These few examples are only aimed at demonstrating typical relationships between the molecular and supramolecular structures of these compounds.

## 5. Degradation of Linear Azaacenes

Azaacenes are more resistant against photolytic and thermal degradations in the presence of oxygen than the corresponding acenes. This is associated with their significantly higher IP values. Detailed studies of the degradation of naphtho[2,3-*g*]quinoxalines and pyrazino[2,3-*b*]phenazines showed that the degradation starts by oxidation of three rings to their peroxide form and then their further transformation to quinones [75]. The terminal ring turned out to be the most resistant in this process whereas the degradation process started by oxidation of the ring adjacent to it.

Degradation in oxygen-free conditions was significantly slower and proceeded through the formation intermolecular covalent bonds resulting in dimers partially losing their aromatic character [75].

## 6. Azaacenes Containing Pyrene-Type Units

**Figure 14:**
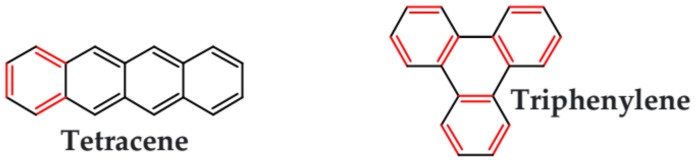
Clar’s sextets in triphenylene and tetracene.

As stated above, linear azaacenes of higher molecular mass and low N/C may undergo degradative oxidation although they are more resistant against this process than the corresponding acenes. Oxidative degradation occurs less readily in non-linear and more isotropic in shape azaacenes. This can be rationalized through Clar’s aromatic sextet rule. According to it, those polyaromatic compounds are more aromatic and, by consequence, more stable in which more π-sextets (benzoid-type rings) can be identified. A comparison of tetracene with triphenylene can be instructive here. In the former only one Clar’s sextet can be distinguished whereas in the latter—three sextets, imposing its higher stability (see Figure 14).

**Figure 15:**
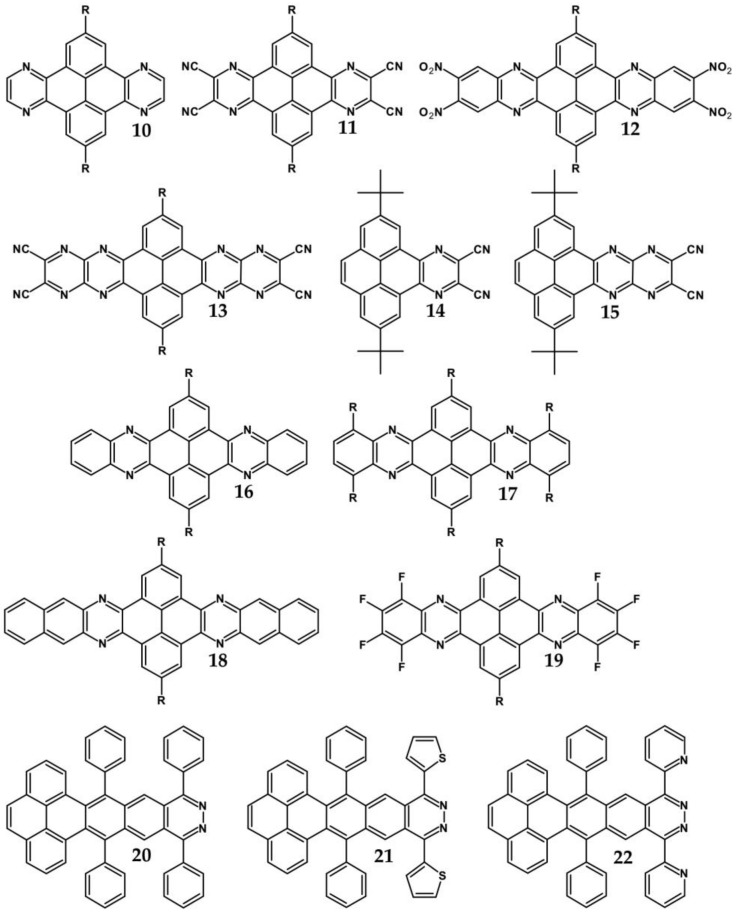
Selected azaacenes containing one pyrene-type units: **10** [76], **11** [76], **12** [76], **13** [76], **14** [77], **15** [77], **16** [76], **17** [78], **18** [78], **19** [78], **20** [79], **21** [79], **22** [79]. R—(tripropan-2-ylsilyl)ethynyl group introduced to facilitate the deposition of these organic semiconductors on silicon substrates.

**Figure 16:**
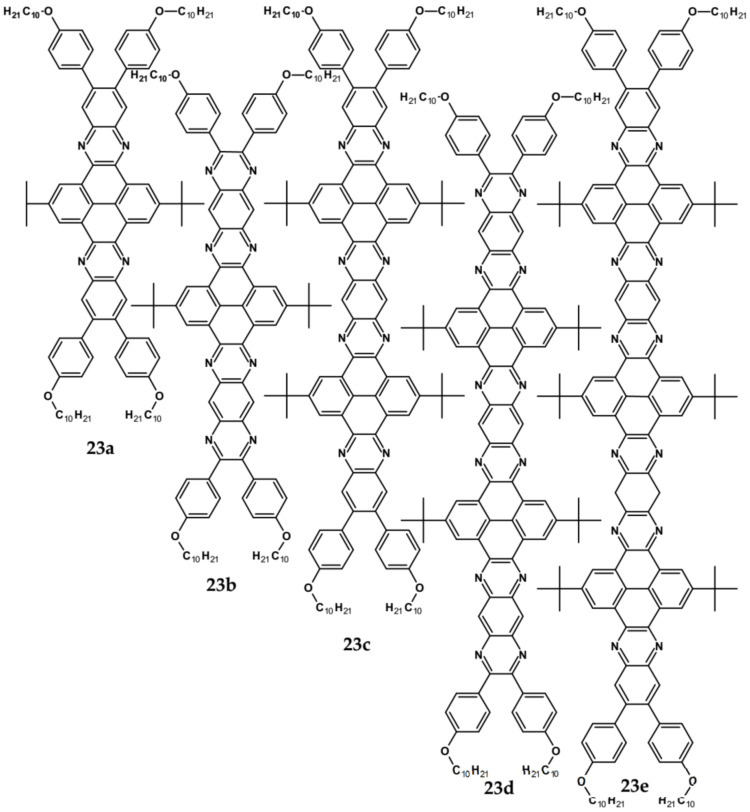
Azaacenes containing increasing number of pyrene-type units [80].

From this point of view azaacenes containing fused pyrene-type units seem very promising. As in the case of linear azaacenes, their properties can be tuned by changing the number of aromatic rings, N/C ratio or by introducing electron accepting/electron donating substituents. Structural formulae of selected azaacenes of this type are presented in Figure 15 and Figure 16 whereas their spectroscopic and electrochemical data are collected in Table 6.

Trends observed for this family of azaacenes are similar to those reported for linear azaacenes. The effect of chemical constitution on their electrochemical properties can be briefly summarized as follows: (i) they undergo one or two or even multistep quasi-reversible reduction, the number of redox couples depending on the N/C ratio; (ii) their first reduction potential rises with increasing number of aromatic rings as well as with increasing N/C ratio; (iii) introduction of electron accepting groups to the terminal ring results in the same effect; (iv) although the first reduction potential (E_red1_) and by consequence electron affinity (EA) almost strictly follow this trend, correlation between E_red1_ and the optical band gap (E_g opt_) is not always evident (see Table 6). This means that electron accepting substituents may affect the positions of the LUMO and HOMO levels in a similar manner showing no significant narrowing of the band gap.

The above outlined trends can be illustrated by comparing the first reduction potentials of **16**, **11** and **12**. These three azaacenes consist of the same core. **16** contains no electron accepting substituents whereas in the remaining two (**11** and, **12**) the terminal ring was functionalized with different electron withdrawing groups (Figure 15). Introduction of these groups significantly rises the reduction potential, the observed shift depending on the substituent capability of lowering the electron density in the core. Two-fold increase of the number of nitrogen atoms while retaining the same number of aromatic rings and the same substituents results in an increase of the first reduction potential (E_red1_) by ca. 550 mV (compare the case of **11** and **13**). The same trend is observed for pyridazine derivatives (**20**, **21**, **22**). Their E_red1_ increases with increasing electron accepting character of the substituent: phenyl < thienyl < pyridyl.

Azaacenes discussed so far contained one pyrene-type unit. There exist, however, reports on azaacenes comprising two or even more such units [80]. In Figure 16 azaacenes of increasing length and increasing number of pyrene units are presented, starting from tetraazahexacene with one pyrene unit and ending up with dodecaazahexadecacene containing three pyrene units (**23a–e**). One of the characteristic features of this group of compounds is a rather weak dependence of their first reduction potential on the N/C ratio and the number of aromatic rings, observed for azaacenes longer than tetraazahexacene (**23a**). In fact, extension of the molecule length from octaazaoctacene (**23b**) to dodecaazahexadecacene (**23e**) results in an increase of E_red1_ by 60 mV, only. The number of redox couples in compounds **23a**, **23b**, **23c**, **23d** and **23e** is equal to the number of azaacene units separated by pyrene units.

As already stated, in the discussed compounds quinoxaline and pyrazinoquinoxaline segments are separated by pyrene segments. Their UV-vis-NIR spectra of strongly vibronic nature are typical of azaacenes with some contribution from pyrene. The band ascribed to 0-0 transition of the shortest compound (**23a**) is hypsochromically shifted with respect to the corresponding bands of the four other compounds by 60 to 90 nm.

## 7. Spatially Extended Azaacenes

**Figure 17:**
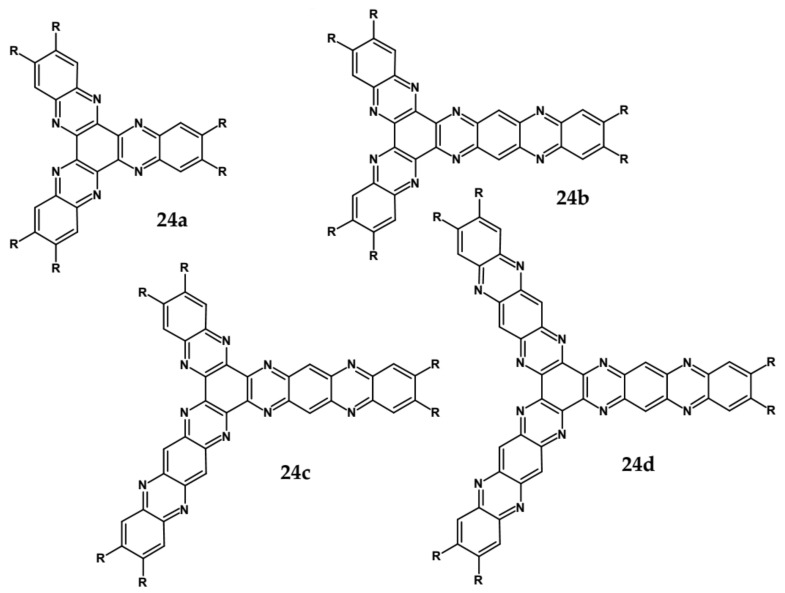
Chemical structures of star-shaped azaacenes. R = *p*-decyloxyphenyl group [81].

Star-shaped azaacenes are typical examples of this group of compounds. In Figure 17 star-shaped azaacenes containing three arms of equal or non-equal length are depicted.

**Figure 18:**
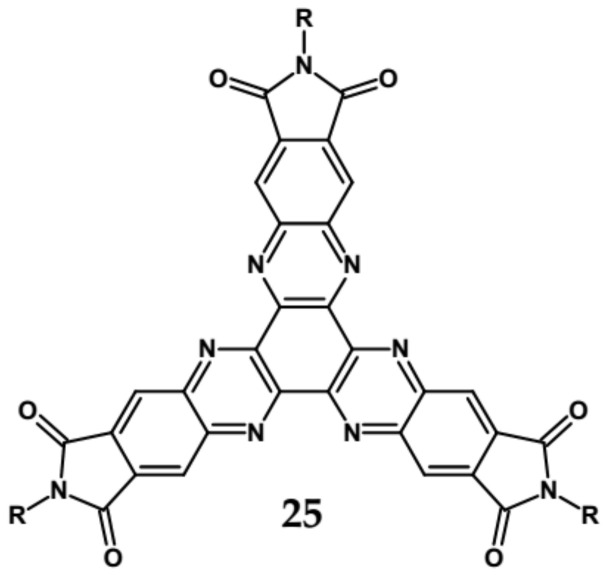
Chemical structure of star-shaped **25** [83]. R = dodecane group.

An attractive feature of this type of azaacenes is the possibility of individual arms engineering [81,82]. Transformation of one arm from quinoxaline-type into pyrazinoquinoxaline results in a significant increase of the first reduction potential and a bathochromic shift of the band ascribed to the 0–0 transition by nearly 40 nm with concomitant increase of E_red1_ from −1.01 V to −0.71 V (compare **24a** and **24b** in Table 7) [81]. Consecutive extension of the second and the third arm brings further increase of E_red1_ and increasing bathochromic shift of the band ascribed to the 0–0 transition (Table 7). Engineering of an individual arm transfers the induced changes to the remaining two arms because the molecule as a whole is aromatic. Finally, functionalizing the terminal ring of quinoxaline-type arms with dicarboxyimide groups (see Figure 18) results in an increase of E_red1_ to −0.91 V and simultaneous increase of the band gap to 2.95 eV [83]. This unexpected finding indicates that lowering of the LUMO level as a result of introducing electron-withdrawing groups was overcompensated by even more pronounced lowering of the HOMO level.

**Figure 19:**
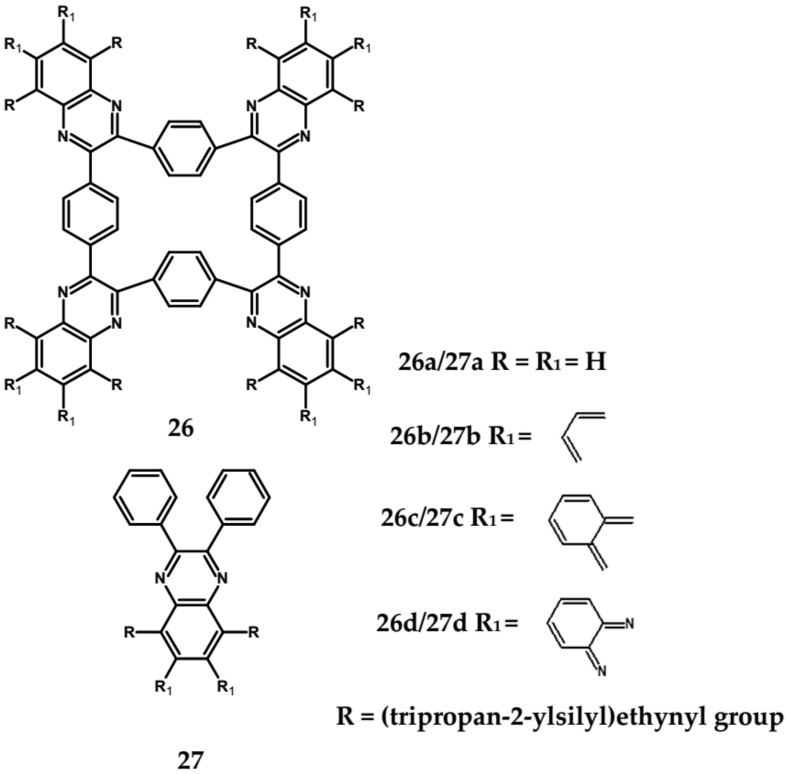
Azaacenes with benzenacyclooctaphane central units [84].

Spatially extended azaacenes containing cyclic, conjugated central unit deserve a special interest [84,85]. In Figure 19 chemical structures of a series of azaacenes of this type, differing in the nature of their azaacene arms are presented: quinoxaline (**26a**), benzoquinoxaline (**26b**) naphthalenequinoxaline (**26c**), pyrazinophenazine (**26d**). Spectroscopic and electrochemical properties of these compounds were compared to those determined for molecules mimicking their arms (**27**) [84].

Spectroscopic properties of **26 a**, **b**, **c**, **d** compounds were very similar to those of the corresponding **27 a**, **b**, **c**, **d** ones, showing only a small bathochromic shift of their absorption and emission bands (ca. 10–15 nm) (see Table 8) [84]. Electrochemical data were somehow surprising. With the exception of **26a** and **27a** for which an increase of E_red_ by 350 mV was observed in the star-like compound, in the three remaining cases a small decrease of E_red_ by 60 to 100 mV was noticed for **26 b**, **c**, **d** as compared to **27 b**, **c**, **d**. This is in contrast to common cases showing that the extension of the pi-system in polyconjugated molecules results in an increase of E_red_. Evidently the central ring does not transmit the conjugation between individual arms.

**Figure 20:**
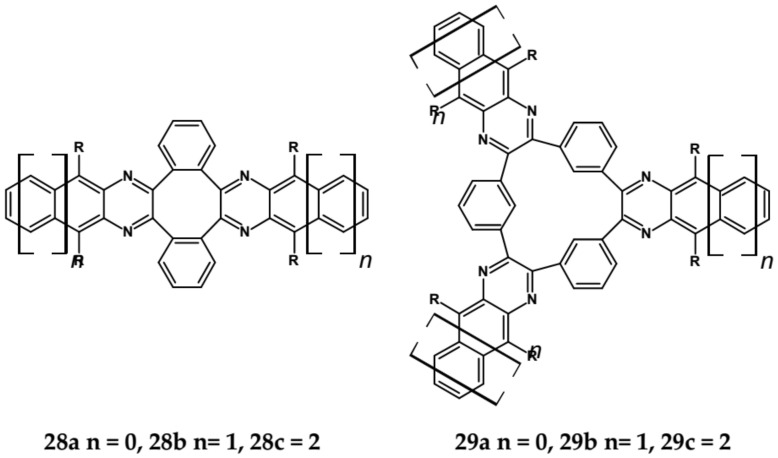
Molecular structure of propeller-shaped azaacenes [85]. R = (triisopropylsilyl)ethynyl group.

Molecules depicted in Figure 20 can be considered as propeller-shaped azaacenes. The propeller arms consist of quinoxaline, benzoquinoxaline and naphthalenequinoxaline units held together through *o*-phenylene (**28 a**, **b**, **c**) or m-phenylene (**29 a**, **b**, **c**) linkers. Spectroscopic properties of both types of azaacenes are similar. Their 0–0 transition band is strongly dependent on the length of the arm, undergoing a bathochromic shift with increasing number of aromatic rings. The conjugation between particular arms via phenylene linkers is rather weak since the 0–0 transition bands in **28 a**, **b**, **c** and **29 a**, **b**, **c** were only slightly bathochromically shifted with respect to the corresponding bands of compounds mimicking their arms (see Table 9).

These spectroscopic findings were corroborated by electrochemical investigations indicating a very modest increase of the first reduction potential in the propeller-shaped compounds as compared to those determined for molecules mimicking their arms (Table 9).

## 8. Nonlinear Azaacenes

**Figure 21:**
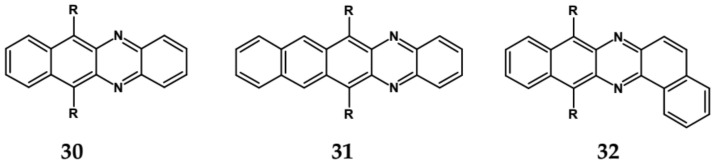
Chemical structures of: 6,11-bis((triisopropylsilyl)ethynyl)benzo[*b*]phenazine (**30**) and 6,13-bis-((triisopropylsilyl)ethynyl)naphtho[2,3-*b*]phenazine (**31**) and L-shaped 8,13-bis[(triisopropylsilyl)ethynyl]dibenzo[*a*,*i*]phenazine (**32**) [88]. R = (triisopropylsilyl)ethynyl group.

Nonlinearity of azaacene molecules results in an increased number of Clar’s sextets as compared to their linear analogues, leading to improved stability. There are several types of nonlinear azaacenes [86,87]: L-shaped, Z-shaped, U-shaped spatially extended of perylene- or pyrene-type and others. The effect of nonlinearity on spectroscopic properties of azaacenes can be evidenced by comparing L-shaped diazapentacene with its linear analogue and linear diazatetracene (see Figure 21). Their spectroscopic data are collected in Table 10. It is evident that nonlinearity induces a strong hypsochromic shift of the band corresponding to the 0–0 transition by nearly 150 nm. E_g opt_ of **32** is even larger than that of linear diazatetracene—**31** [88].

**Figure 22:**
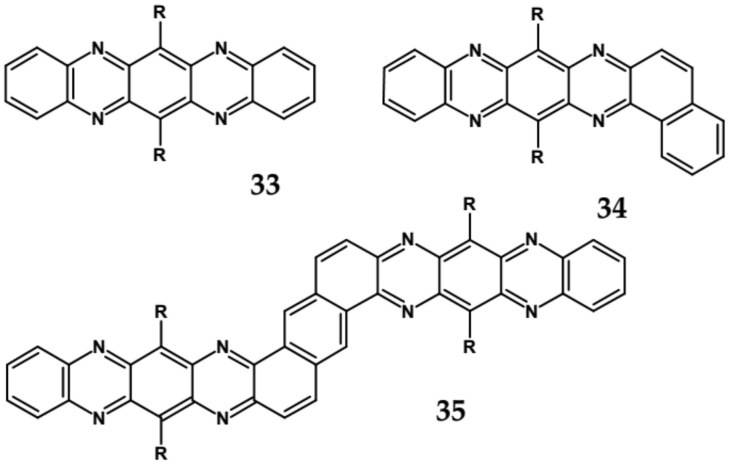
Molecular formulae of linear tetraazapentacene (6,13-bis((triisopropylsilyl)ethynyl)-5,7,12,14-tetraazapentacene) **33**, L-shaped tetraazahexacene (8,15-bis((triisopropylsilyl)ethynyl)benzo[a]quinoxalino[2,3-i]phenazine) **34** and Z-shaped 6,12,19,25-tetra((triisopropylsilyl)ethynyl)quinoxalino 2,3-i]quinoxalino [2′,3″:6′,7′]quinoxalino[2′,3′:5,6]nafto[2,3-a]phenazine **35** [89]. R = (triisopropylsilyl)ethynyl group.

Comparison of spectroscopic data obtained for linear **33**, L-shaped **34** and Z-shaped **35** azaacenes (Figure 22 and Table 10) shows that the 0–0 transition band of **33** is bathochromically shifted by only 31 nm with respect to the corresponding band in linear diazapentacene **34**. Similarly, its E_gopt_ is only slightly narrower by 0.06 eV as compared to E_g_ of **34**. This finding should be considered as a spectroscopic evidence of a very weak conjugation between tetraazapentacene linear segments of this Z-shaped molecule.

Diazaacenes usually exhibit one quasi-reversible redox couple at negative potentials with respect to Fc/Fc^+^. In Table 10 reduction potentials of L-shaped diazapentacene **32**, linear diazapentacene **31** and linear diazatetracene **30** are compared. The lowest reduction potential is observed for the L-shaped compound, which is even lower than that measured for the linear one **30**. This can be considered as a manifestation of less efficient conjugation of **32** due to the presence of two mutually independent Clar’s sextets.

Tetraazaacenes, such as linear tetraazapentacene **33**, L-shaped tetraazapentacene **34** as well as Z-shaped octaazaundecacene **35**, usually exhibit two redox couples at negative potentials, ascribed to two consecutive one-electron reductions to a radical anion and dianion, respectively. Furthermore, in this case the L-shaped molecule shows lower reduction potential than the linear one, although the latter is shorter by one ring. Significant increase of the first reduction potential is observed for the Z-shaped compound **35**. This molecule consists of two linear tetraazapentacene segments connected by an aromatic ring. An increase of the potential by 0.17 V with respect to that of linear tetraazapentacene **33** indicates that the conjugation extends over the connecting (central) aromatic ring.

**Figure 23:**
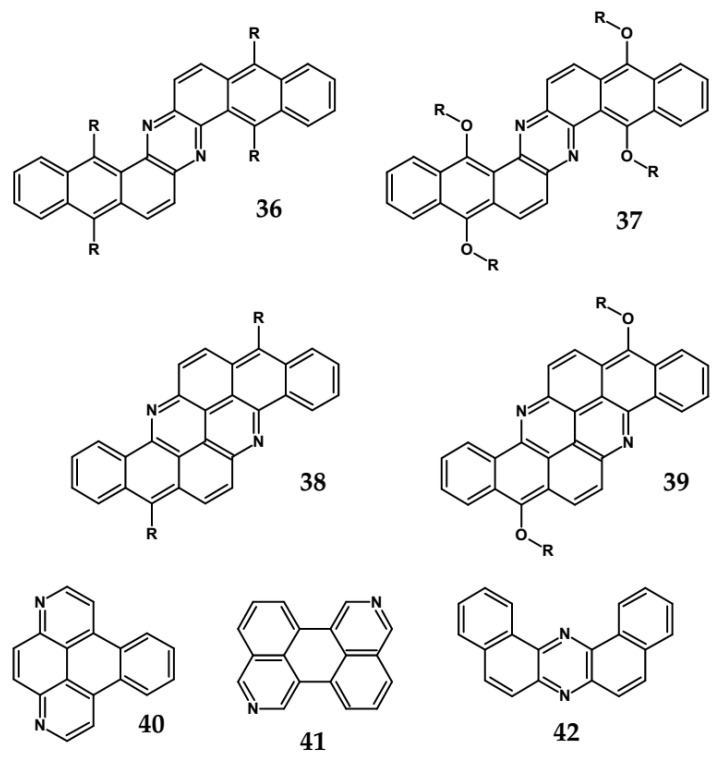
Azaacenes **36** [63], **37** [60], **38** [90], **39** [61], **40** and **41** [91], **42** [64]. R_1_ and R_3_ = (triisopropylsilyl)ethynyl, R_2_ = octyl group.

Solution processable Z-shaped azaacenes which can be considered as derivatives of dinaphtho[2,3-*a*:2′,3′-*h*]phenazine (see Figure 23) were synthesized by Goto et al. [63] and Kotwica et al. [37,38]. Alkoxy groups introduced to **37** induce solution processability but in addition, due to their electron donating properties, they lower the band gap which is slightly lower than the gap of **36** and significantly lower that the gap of **42** – unsubstituted dibenzophenazine (see Table 11). **38** and **39**, i.e., benzo[*h*]benz[5,6]acridino[2,1,9,8-*klmna*]acridines were reported by Kotwica et al. [61]. These compounds can be considered as two L-shaped azatetracenes fused together. Note a close similarity of the absorption and emission spectra as well as the reduction potential of **37** and **39**. **40** exhibits a large gap and a very low reduction potential. Consistent with these finding it emits ultraviolet radiation.

In summary, the possibilities of precise tuning of azaacenes properties should be pointed out, which are more numerous as compared to acenes. Electronic, optical and redox properties can be modified not only by increasing the number of aromatic rings but also by changing N/C ratio and the mode of nitrogen atoms distribution within the molecule. Finally, changing the shape of molecules (linear vs. L-shaped, Z-shaped, U-shape, condensed etc.) not only leads to alteration of their redox properties but also may result in distinctly different supramolecular organizations. Data reported in reference [92] should be considered as an instructive example of such tuning of properties. 6,13-bis((triisopropylsilyl)ethynyl)-1,4-diazapentacene, i.e., diazapentacene containing two nitrogen atoms in its terminal ring shows hole-only electrical transport in the field effect transistor configuration with a mobility of 1.2 cm^2^V^−1^s^−1^. Transferring these two nitrogen atoms to one of the inner rings as in the case of 6,13-bis((triisopropylsilyl)ethynyl)-5,14-diazapentacene transforms this hole transporting semiconductor into an ambipolar one. Once again, in the FET configuration values of 0.22 cm^2^V^−1^s^−1^ and 1.1 cm^2^V^−1^s^−1^ were measured for electron and holes mobilities, respectively. Increasing the number of nitrogen atoms from two to four gives rise to a purely electron transporting (n-type) semiconductor, exhibiting electron mobility of 3.3 cm^2^V^−1^s^−1^ [92].

Specific properties, including processability, can also be tuned by functionalization. Functionalization of dinaphtho[2,3-*a*:2′,3′-*h*]phenazine with alkoxy groups [60] lowers the band gap of this electroluminophore and increases its solubility. Additionally, strong dependence of the melting temperature on the substituent length is observed. In particular, it decreases from nearly 200 °C to below 100 °C when butoxy substituents are replaced by dodecyloxy ones, with redox and spectroscopic properties remaining essentially the same. This appropriately functionalized dinaphtho[2,3-*a*:2′,3′-*h*]phenazine can be conveniently melt-processed.

## 9. Applications of Azaacenes

High stability of azaacene radical anions and in some cases radical cations facilitates their use as components of organic and hybrid (organic/inorganic) electronic and optoelectronic devices. This is important because in operating conditions organic semiconductors are being temporarily transformed from their neutral form to the ionic one, due to electron or hole injection. Several light emitting diodes of excellent efficiency were fabricated where azaacenes served as electroluminophores in active layers [82,93,94,95] or as electron transporting or hole blocking layers [96]. An important drawback of classical organic light emitting diodes should be pointed out. In these devices electrons and holes injected from the electrodes form excitons. Only 25% of them are singlet excitons which may undergo radiative recombination generating light. 75% are triplet excitons which do not contribute to the generation of light. Thus, the maximum internal quantum efficiency in classical organic diodes cannot exceed 25%. However, if the energy difference between the singlet and the triplet states is small, the singlet state can be thermally repopulated through the reversed intersystem crossing (RISC), transforming inactive triplet excitons into singlet ones. This phenomenon is called “thermally activated delayed fluorescence” (TADF) and is very intensively studied in recent years, because TADF diodes can reach internal efficiencies approaching 100%. Several azaacenes were reported as efficient TADF emitters in recent years, among them substituted dibenzo[*a*,*c*]phenazines **52**, **53** (see Table 12) [97] or 10-(acenaphtho[1′,2′:5,6]pyrazino[2,3-*f*][1,10]phenanthrolin-12-yl)-10*H*-phenoxazine **46** and 7-(acenaphtho[1′,2′:5,6]pyrazino[2,3-*f*] [1,10]phenanthrolin-12-yl)-7*H*-benzo[*c*]phenoxazine **47** (Table 12) [98]. Table 12 presents chemical formulae of azaacenes used as components of OLEDs together with selected parameters of the fabricated test devices (positions **43**–**64**).

In recent years, in the domain of organic solar cells, significant research effort was directed towards the design and fabrication of “all organic” solar cells in which fullerene derivative-type acceptors, typically used in “bulk heterojuncion” devices, were replaced by purely organic acceptors (see for example [99,100]). Various azaacenes were tested as acceptors in such devices [36,37,101]. Formulae of selected azaacene-type acceptors are listed in Table 12 (positions **65**–**68**), together with parameters of the test devices.

In the past decade, devices in which the active layer consists of various derivatives of lead or tin halides of perovskite structure, the so called “perovskite solar cells”, dominated the research on hybrid (inorganic/organic) photovoltaics [102], despite several problems connected to their stability in operating conditions. However, significant effort was achieved in few recent years in this respect, as far as elucidation of degradation mechanisms and stability improvements are concerned [103]. Nitrogen atoms-rich azaacene derivatives are very well suited to serve as electron transporting layers in perovskite solar cell [104,105,106]. Chemical formulae of azaacenes used in perovskite-type devices are presented in Table 12 (positions **69**–**74**) together with selected device parameters.

As already mentioned, electrical transport properties of azaacenes depend on four major factors: N/C ratio, the number of aromatic rings, the presence of electron donating (electron accepting) substituents and finally the shape of the molecule—linear vs. L-shaped, Z-shaped, U-shaped etc. Several azaacenes were used as active components of n-channel field effect transistors (FETs), some of them are shown in Table 12 (**80** [107]). Azaacenes of lower N/C can be ambipolar, i.e., transporting either holes or electrons depending on polarity. Some ambipolar azaacenes are depicted in Table 12 (**79** [108], **78** [109]). Azaacenes exhibiting only p-type electrical transport and for these reasons used in p-channel FETs were also reported (**86** in Table 12) [110]. In Table 12 (positions **75**–**88**) chemical formulae of selected azaacenes are shown tested as components of active layers in field effect transistors together with their charge carriers mobilities measured in the FET configuration. It should be noted that tetracholorotetraazapentacene reported in [111] exhibited extremely high electron mobility of ca. 28 cm^2^ V^−1^ s^−1^.

Azaacenes are also used in organic phototransistors, i.e, devices converting light to current, somehow resembling photodiodes with an amplifier transistor connected. Test phototransistors of reproducible parameters were fabricated from 6,8,15,17-tetraaza-1,18,4,5,9,10,13,14-tetra-benzoheptacene (**91**) (see Table 12) [112].

Application of azaacenes as components of organic memory devices of WORM (write-once-read-many-times) or FLASH (storage that can be electrically erased and reprogrammed) have drawn a significant research interest in recent years. Test devices described in [29] exhibited improved stability both in neutral states as well as in on states of reduced resistivity. Selected azaacenes used as components of organic memory devices are presented in Table 12 (**92–95**).

To summarize, azaacenes exhibit several interesting properties difficult to match by other families of electroactive organic compounds. Their redox, luminescent and electrical transport properties can be precisely tuned by changing the number of aromatic rings being in conjugation, N/C ratio and nitrogen atoms distribution. Further modifications are possible by introducing electron accepting or electron donating substituents. Their solution or melt processability can be induced or improved by attaching appropriate side groups which, if properly designed, do not worsen their precious electrical transport properties. Thus, it is clearly evident that further development of azaacene-based materials has to be inherently associated with concomitant progress in the synthesis of new functional azaacene derivatives with functions intentionally designed for a given application. This is a very promising direction, taking into account quick progress in heterocyclic chemistry with elaboration of new catalytic systems leading to significantly simplified reaction pathways and resulting in improved yields. One direction in the design of new azaacenes seems extremely interesting, although, so far, little explored. This is self-assembly through molecular recognition, i.e., bio-inspired approach. Introducing substituents capable of forming multiple, parallel intermolecular hydrogen bonds should facilitate formation of ordered monolayers on appropriate substrates such as HOPG or gold. Moreover, appropriate bio-inspired functionalization may facilitate controlled layer by layer deposition of these semiconductors, governed by molecular recognition [139]. Nanoprocessing of this type is of crucial importance for further development of organic nanodevices.

## 10. Conclusions

This short review demonstrates that azaacenes are extremely interesting compounds for electronic and optoelectronic applications. They are in general more stable and more resistant to degradative oxidation than the corresponding acenes. Moreover, by changing their N/C ratio as well as distribution mode of N-atoms in the molecule their optical, electronic and redox properties can be tuned in a wide range. Additional modification of their properties can be achieved by changing the molecular shape (linear vs. L-shaped, Z-shaped, U-shaped, fused, etc.) and by introduction of substituent of electron accepting or electron donating character. Finally, they can be rendered solution and melt processable by attaching appropriate flexible solubilizing groups to the conjugated core or substituents lowering the melting temperature. For all these reasons various types of azaacenes are studied as active components of field effect transistors, phototransistors, light emitting diodes, memory devices and others.

## Figures and Tables

**Table 1:** Reduction potentials, electron affinities and optical band gaps of selected acenes and their azaacene analogues.

Compound	E_1/2red_^#^ vs. Fc/Fc^+^ [V]	EA * [eV]	E_g opt_ [eV]
**Acenes**			
Naphthalene	−3.44 [13]	0.77	4.14 [14]
Anthracene	−2.91 [13]	1.40	3.20 [14]
**Azaacenes**			
Quinoline (1N)	−2.77 [15]	1.56	3.94 [16] **
Quinoxaline (2N)	−2.26 [15]	2.16	3.85 [17] **
Acridine (1N)	−2.26 [15]	2.16	3.22 [18] **
Phenazine (2N)	−1.83 [15]	2.67	3.07 [19] **
Pyrazinoquinoxaline (4N)	−1.60 [20]	2.94	2.95 [21] **

^#^ recalculated from original data in which potential was given vs. SCE. * EA calculated by following the procedure proposed in [22], ** E_opt_ estimated from the absorption spectra shown in reference [16,17,18,19,21] by determining the wavelength of the absorption onset and converting it from nm to eV.

**Table 2:** Electrochemical and optical parameters of series 1 and series 2 diazaacenes [69].

Compounds	E_1/2red_ vs. Fc/Fc^+^ [V]	EA * [eV]	E_g opt_ [eV]
**1a**	−1.1	3.53	2.38
**1b**	−0.8	3.89	1.84
**1c**	−0,7	4.00	1.49
**2a**	−1.1	3.53	2.20
**2b**	−0.8	3.89	1.69
**2c**	−0.48	4.26	1.35

* EA calculated following the procedure proposed in [22].

**Table 3:** Spectroscopic properties of di- and tetraazaacenes halogenated in their terminal ring, registered for hexane solution (series 3 and 4) [50] and dichloromethane solutions (series 5) [71].

Compound	λ_max abs_ [nm]	λ_max emi_ [nm]	Stokes Shift [nm]
**3a**	572	577	5
**3b**	602	616	14
**3c**	620	627	7
**4a**	548		
**4b**	574		
**4c**	598		
**5a**	586	590	4
**5b**	602	643	44
**5c**	611	657	42

**Table 4:** Spectroscopic and electrochemical data of selected halogen derivatives of tetraazapentacenes [72], tetraazahexacenes [51] and tetraazapentacene with nitro substituent [43].

Compound	E_g opt_ [nm]/[eV]	E_red_ vs. Fc/Fc^+^ [V]	EA * [eV]
**6a**	737/1.68	−0.60	4.12
**6b**	752/1.65	−0.60	4.12
**6c**	744/1.67	−0.70	4.00
**7a**	862/1.44	−0.55	4.18
**7b**	1039/1.19	−0.48	4.26
**7c**	1000/1.24	−0.53	4.20
**8a**	780/1.59 **	−0.52	4.22
**8b**	867/1.43 **	−0.27	4.51

* EA calculated by following the procedure proposed in [22], ** E_opt_ optical band gaps were calculated from the UV-vis-NIR spectra by determining the wavelength of the absorption onset and converting it from nm to eV.

**Table 5:** Spectroscopic and electrochemical properties of azaacenes of increasing number of aromatic rings containing triphenylamine substituents [73]. E_ox_ vs. Fc/Fc^+^.

Compounds	λ_max abs_ [nm]	E_g opt_ [eV]	E_ox1_ [V]	E_ox2_ [V]
**9a**	413	2.60	0.26	0.48
**9b**	493	2.17	0.24	0.42
**9c**	455	2.36	0.25	0.44
**9d**	560	1.88	0.22	0.37

**Table 6:** Spectroscopic and electrochemical data of selected azaacenes containing pyrene-type units [76,77,78,79,80].

Compounds	λ_max abs_ [nm]	E_g opt_ [eV]	E_red_ vs. Fc/Fc^+^ [V]	EA *** [eV]	Compounds	λ_max abs_ [nm]	E_g opt_ [eV]	E_red_ vs. Fc/Fc^+^ [V]	EA *** [eV]
**10**	417	2.81	−0.79	3.90	**19**	485	2.42	−1.51 *	3.05
**11**	435	2.76	−0.76	3.93	**20**	441	2.51	−1.69 **	2.84
**12**	440	2.70	−0.73	3.97	**21**	471	2.37	−1.54 **	3.01
**13**	482	2.37	−0.21	4.58	**22**	464	2.41	−1.62 **	2.92
**14**	455	2.09	−1.42 *	3.15	**23a**	443	2.65	−1.68 **	2.85
**15**	571	1.85	−0.71 *	3.99	**23b**	500	2.34	−1.22 **	3.39
**16**	421	2.88	−1.73 *	2.79	**23c**	525	2.25	−1.19 **	3.43
**17**	436	2.69	−1.57 *	2.98	**23d**	529	2.22	−1.16 **	3.46
**18**	425	2.78	−1.44 *	3.13	**23e**	538	2.18	−1.14 **	3.48

* recalculated from original data in which potential was given vs. SCE, ** recalculated from original data in which potential was given vs. Ag/AgCl, *** **EA** calculated following the procedure proposed in [22].

**Table 7:** Spectroscopic and electrochemical data of star-shaped azaacenes **24** [81], **25** [83].

Compounds	λ_max abs_ [nm]	λ_max emi_ [nm]	E_g opt_ [eV]	E_red_ vs. Fc/Fc^+^ [V]	EA * [eV]
**24a**	471	552	2.43	−1.01	3.64
**24b**	508	593	2.23	−0.71	3.99
**24c**	530	605	2.13	−0.60	4.12
**24d**	543	615	2.08	−0.53	4.20
**25**	399	504	2.95	−0.91	3.90

* EA calculated by following the procedure proposed in [22].

**Table 8:** Spectroscopic and electrochemical data of azaacenes containing conjugated cyclic central unit [84].

Compounds	λ_max abs_ [nm]	λ_max emi_ [nm]	E_g opt_ [eV]	E_red_ vs. Fc/Fc^+^ [V]	EA * [eV]
**26a**	378	422	2.99	−1.50	3.90
**26b**	497	508	2.42	−1.63	3.06
**26c**	597	604	1.98	−1.50	2.91
**26d**	595	607	2.01	−1.09	3.06
**27a**	369	427	3.06	−1.85	3.54
**27b**	485	497	2.47	−1.56	2.65
**27c**	582	590	2.07	−1.40	2.99
**27d**	586	599	2.05	−1.03	3.18

* EA calculated by following the procedure proposed in [22].

**Table 9:** Spectroscopic and electrochemical date of propeller-shaped azaacenes of **28** and **29** series [85].

Compounds	λ_max abs_ [nm]	λ_max emi_ [nm]	E_g opt_ [nm]/[eV]	E_red_ vs. Fc/Fc^+^ [V]	EA * [eV]
**28a**	391	413	409/3.03	−1.58	2.97
**28b**	490	500	504/2.46	−1.55	3.00
**28c**	589	594	603/2.06	−1.33	3.26
**29a**	388	433	411/3.02	−1.82	2.68
**29b**	493	506	510/2.43	−1.57	2.98
**29c**	596	601	610/2.03	−1.34	3.25

* **EA** calculated by following the procedure proposed in [22].

**Table 10:** Spectroscopic data, redox potentials and electron affinities (EA) of linear, L-shaped and Z-shaped azaacenes [88,89].

Compound	λ_max abs_ [nm]	λ_max emi_ [nm]	E_g opt_ [nm]/[eV]	E_red_ vs. Fc/Fc^+^ [V]	EA * [eV]
**30**	570	577	585/2.12	−1.23 **	3.38
**31**	693	699	709/1.75	−1.05 **	3.59
**32**	544	553	558/2.22	−1.79 **	2.72
**33**	681	694	−/1.94	−0.68	4.03
**34**	653	699	−/1.75	−0.76	3.93
**35**	712	688	−/1.75	−0.51	4.23

* EA calculated by following the procedure proposed in [22] ** E_red_ calculated from data reported in [88].

**Table 11:** Spectroscopic and electrochemical data of azaacenes **36** [63], **37** [60], **38** [90], **39** [61], **40** and **41** [91], **42** [64].

Compounds	λ_max abs_. [nm]	λ_max emi_ [nm]	E_g opt_ [eV]	E_red_ vs. Fc/Fc^+^ [V]	EA [eV]
**36**	499	501	-	−1.20	3.41
**37**	493	515	2.40	−1.63	2.91
**38**	554	-	2.23	-	-
**39**	515	524	2.33	−1.62	2.92
**40**	369	370	4.11	−2.02	2.45
**41**	423	431	3.18	−1.77	2.74
**42**	416	425	-	−1.88	2.61

**Table 12:** Structures of azaacenes and their application in devices.

Compound	Device’s Properties
**Emitters in organic light emitting diode**	
**43** 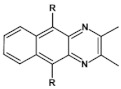	maximum luminance 1000 cd m^−2^ * efficiency 5 cd A^−1^ EQE = 0.53%, **λ_max emi_** = 540 nm * [93]
**44** 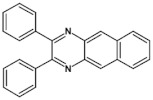	maximum luminance 2321 cd m^−2^ efficiency 0.79 cd A^−1^, **λ_max emi_** = 547 nm [85]
**45** 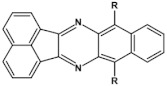	maximum luminance 5792 cd m^−2^ * efficiency 2.88 cd A^−1^ **λ_max emi_** = 502 nm [113]
**46** 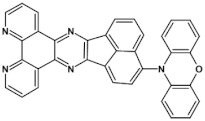	maximum luminance > 600 cd m^−2^ * efficiency 5.4 cd A^−1^ EQE = 7.2%, **λ_max emi_** = 640 nm [98]
**47** 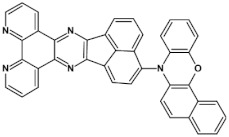	maximum luminance > 300 cd m^−2^ * efficiency 0.8 cd A^−1^ EQE = 2.0%, **λ_max emi_** = 665 nm [98]
**48** 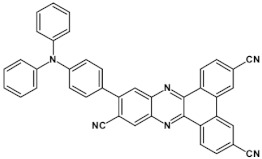	maximum luminance > 300 cd m^−2^ * EQE = 22.80%, **λ_max emi_** = 698 nm [114]
**49** 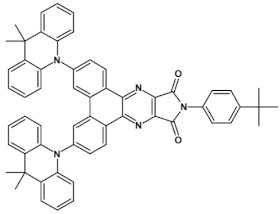	maximum luminance > 10,000 cd m^−2^ EQE = 26.00%, [115]
**50** 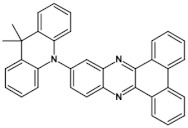	efficiency 51.8 cd A^−1^ EQE = 21.8%, **λ_max emi_** = 577 nm [116]
**51** 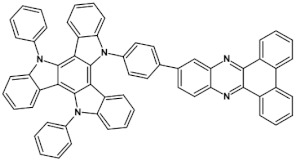	efficiency 47.1 cd A^−1^ EQE = 23.8%, **λ_max emi_** = 587 nm [116]
**52** 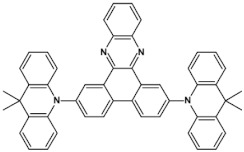	maximum luminance > 6000 cd m^−2^ * EQE = 19.4%, **λ_max emi_** = 580nm [97]
**53** 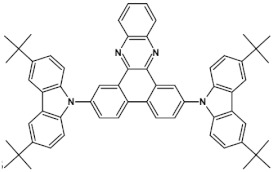	maximum luminance > 10,000 cd m^−2^ * EQE = 22.1%, **λ_max emi_** = 547 nm [97]
**54** 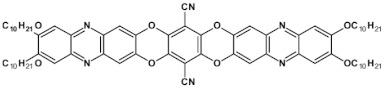	maximum luminance > 1000 cd m^−2^ * EQE = 3.3%, **λ_max emi_** = 536 nm phosphorescence [33]
**55** 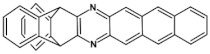	maximum luminance 8600 cd m^−2^, EQE = 0.33%, **λ_max emi_** = 511 nm [117]
**56** 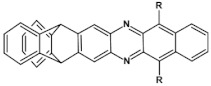	maximum luminance > 200 cd m^−2^, efficiency 1 cd A^−1^, **λ_max emi_** = 590 nm [70]
**57** 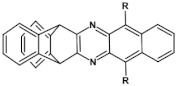	maximum luminance 593 cd m^−2^ *, efficiency 0.85 cd A^−1^, **λ_max emi_** = 502 nm [118]
**58** 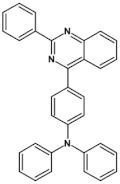	maximum luminance 20780 cd m^−2^, EQE = 3.1%, **λ_max emi_** = 500 nm [119]
**59** 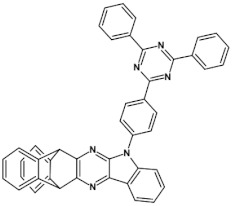	maximum luminance > 500 cd m^−2^ *, efficiency 11.6 cd A^−1^, EQE = 10.4%, **λ_max emi_** = 456 nm [120]
**60** 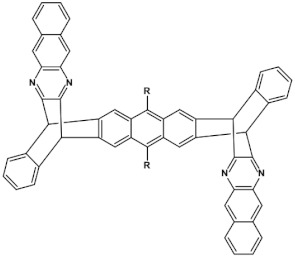	maximum luminance 920 cd m^−2^ *, efficiency 80 cd A^−1^, EQR = 0.53%, **λ_max emi_** = 495 nm [31]
**61** 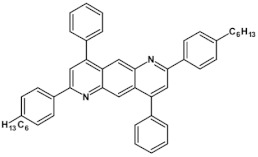	maximum luminance 595 cd m^−2^ *, efficiency 7.0 cd A^−1^, EQE = 2.0%, **λ_max emi_** = 520 nm* [15]
**62** 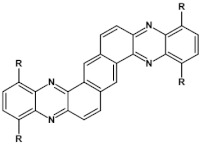	maximum luminance 80 cd m^−2^, **λ_max emi_** = 508 nm [89]
**63** 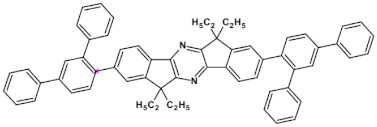	efficiency 2.13 cd A^−1^, EQE = 4.61%, **λ_max emi_** = 440 nm [121]
**64** 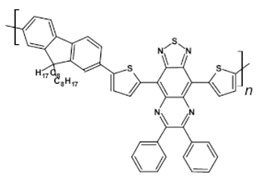	EQE = 0.05%, **λ_max emi_** = 970 nm [122]
**Acceptors for solar cells**	
**65** 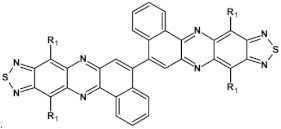	V_OC_ = 0.81 V, FF = 37.3%, J_SC_ = −6,77 mA cm^2^ PCE = 2.03% [101]
**66** 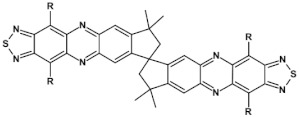	V_OC_ = 0.75 V, FF = 38.3%, J_SC_ = −5.66 mA cm^2^, PCE = 1.62% [35]
**67** 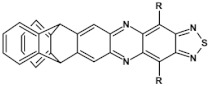	V_OC_ = 0.80 V, FF = 41%, J_SC_ = −6,6 mA cm^2^, PCE = 2.19% [37]
**68** 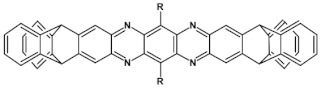	V_OC_ = 0.85 V, FF = 36%, J_SC_ = −7.51 mA cm^2^, PCE = 2.28% [36]
**Electron Transporting Layers for Perovskite Solar Cells**	
**69** 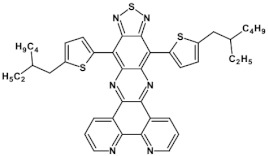	μ_e_ = 2.8 × 10^−4^ cm^2^ V^−1^ s^−1^, μ_e_ = 2.7 × 10^−3^ cm^2^ V^−1^ s^−1^ (100 °C) [104]
**70** 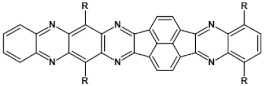	μ_e_ = 4.7 × 10^−4^ cm^2^ V^−1^ s^−1^ [105]
**71** 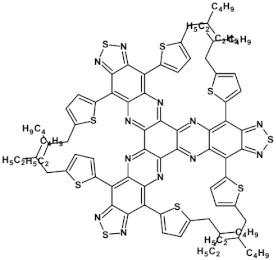	μ_e_ = 1.73 × 10^−2^ cm^2^ V^−1^ s^−1^ [106]
**72** 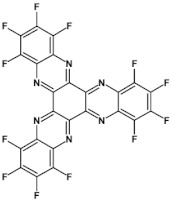	μ_e_ = 1.9 × 10^−4^ cm^2^ V^−1^ s^−1^ [123]
**73** 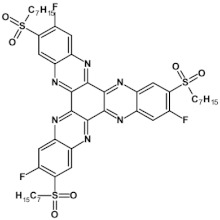	μ_e_ = 5.13 × 10^−3^ cm^2^ V^−1^ s^−1^ [124]
**74** 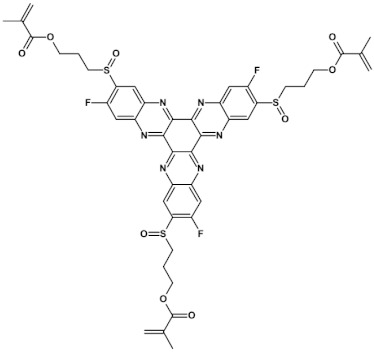	μ_e_ = 1.68 × 10^−3^ cm^2^ V^−1^ s^−1^ (doped by Et_3_N) [125]
**Active layers in organic field effect transistors**	
**75** 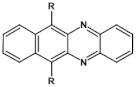	μ_e_ = 7 × 10^−3^ cm^2^ V^−1^ s^−1^ [69]
**76** 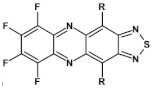	μ_e_ = 7 × 10^−2^ cm^2^ V^−1^ s^−1^ [126]
**77** 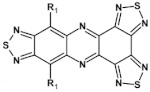	μ_e_ = 9 × 10^−4^ cm^2^ V^−1^ s^−1^ [127]
**78** 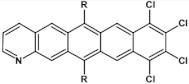	μ_e_ = 0.14 cm^2^ V^−1^ s^−1^, μ_h_ = 0.12 cm^2^ V^−1^ s^−1^ [109]
**79** 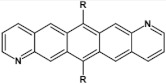	μ_e_ = 0.15 cm^2^ V^−1^ s^−1^, μ_h_ = 0.11 cm^2^ V^−1^ s^−1^ [108]
**80** 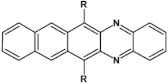	μ_e_ = 0.05 cm^2^ V^−1^ s^−1^, μ_h_ = 4 × 10^−4^ cm^2^ V^−1^ s^−1^ [107]
**81** 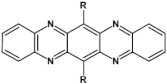	μ_e_ = from 1 do 3.3 cm^2^ V^−1^ s^−1^ (depend of temperatures of substrate—from 25 °C to 100 °C) [107]μ_e_ = 11 cm^2^ V^−1^ s^−1^ (measured in vacuum) [128]
**82** 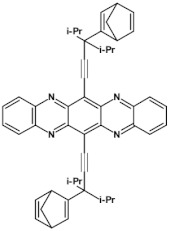	μ_e_ = 3.5 × 10^−4^ cm^2^ V^−1^ s^−1^ [129]
**83** 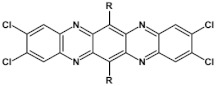	μ_e_ = 27.8 cm^2^ V^−1^ s^−1^ (measured in vacuum) [111]
**84** 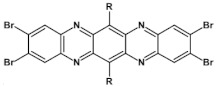	μ_e_ = 0.56 cm^2^ V^−1^ s^−1^ [130]
**85** 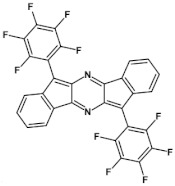	μ_e_ = 3.7 × 10^−2^ cm^2^ V^−1^ s^−1^ [131]
**86** 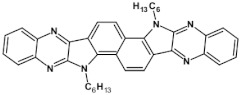	μ_h_ = 0.06 cm^2^ V^−1^ s^−1^ [110]
**87** 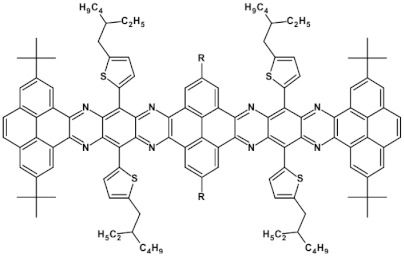	μ_h_ = 8.1 × 10^−3^ cm^2^ V^−1^ s^−1^ [132]
**88** 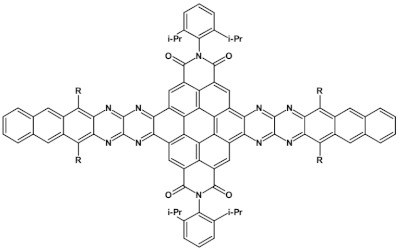	μ_e_ = 8.1 × 10^−4^ cm^2^ V^−1^ s^−1^, μ_h_ = 2 × 10^−4^ cm^2^ V^−1^ s^−1^ [133]
**Self-assembled monolayers (SAM) for functionalization of gold electrodes for** **organic field effect transistors**	
**89** 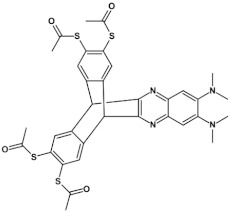	on/off ratio increased from 10^2^ to 10^4^, μ_e_ = from 10^−3^ cm^2^ V^−1^ s^−1^ to 10^−2^ cm^2^ V^−1^ s^−1^ [134]
**Photocatode**	
**90** 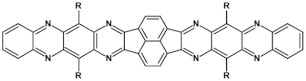	current density 0.13 mA cm^−2^ at −0.13 V (Evs RHE) [135]
**91** 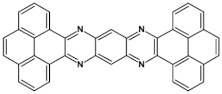	Detected [112]
**Memory device**	
**92** 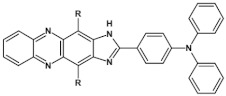	“ON” state a 2.25 V, “OFF” state −0.95 V, 14 cycles of working [136]
**93** 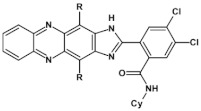	“ON” state a ~3.00 V, “OFF” state −1.65 V *, [137]
**94** 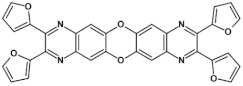	“ON” state a ~2.2 V, “OFF” state −1.2 V * [138]
**95** 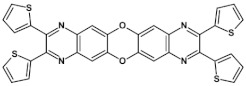	“ON” state a ~1.8 V, “OFF” state −2.0 V * [139]

R = (triisopropylsilyl)ethynyl, * determined from plots.

## Data Availability

Not applicable.
